# A Genome Wide Association Study of *Plasmodium falciparum* Susceptibility to 22 Antimalarial Drugs in Kenya

**DOI:** 10.1371/journal.pone.0096486

**Published:** 2014-05-08

**Authors:** Jason P. Wendler, John Okombo, Roberto Amato, Olivo Miotto, Steven M. Kiara, Leah Mwai, Lewa Pole, John O'Brien, Magnus Manske, Dan Alcock, Eleanor Drury, Mandy Sanders, Samuel O. Oyola, Cinzia Malangone, Dushyanth Jyothi, Alistair Miles, Kirk A. Rockett, Bronwyn L. MacInnis, Kevin Marsh, Philip Bejon, Alexis Nzila, Dominic P. Kwiatkowski

**Affiliations:** 1 Medical Research Council (MRC) Centre for Genomics and Global Health, University of Oxford, Oxford, United Kingdom; 2 Kenya Medical Research Institute (KEMRI)/Wellcome Trust Collaborative Research Program, Kilifi, Kenya; 3 Mahidol-Oxford Tropical Medicine Research Unit, Mahidol University, Bangkok, Thailand; 4 Wellcome Trust Sanger Institute, Hinxton, Cambridge, United Kingdom; 5 Wellcome Trust Centre for Human Genetics, University of Oxford, Oxford, United Kingdom; 6 Department of Biology, King Fahd University of Petroleum and Minerals, Dhahran, Saudi Arabia; University of Melbourne, Australia

## Abstract

**Background:**

Drug resistance remains a chief concern for malaria control. In order to determine the genetic markers of drug resistant parasites, we tested the genome-wide associations (GWA) of sequence-based genotypes from 35 Kenyan *P. falciparum* parasites with the activities of 22 antimalarial drugs.

**Methods and Principal Findings:**

Parasites isolated from children with acute febrile malaria were adapted to culture, and sensitivity was determined by *in vitro* growth in the presence of anti-malarial drugs. Parasites were genotyped using whole genome sequencing techniques. Associations between 6250 single nucleotide polymorphisms (SNPs) and resistance to individual anti-malarial agents were determined, with false discovery rate adjustment for multiple hypothesis testing. We identified expected associations in the *pfcrt* region with chloroquine (CQ) activity, and other novel loci associated with amodiaquine, quinazoline, and quinine activities. Signals for CQ and primaquine (PQ) overlap in and around *pfcrt*, and interestingly the phenotypes are inversely related for these two drugs. We catalog the variation in *dhfr, dhps, mdr1, nhe, and crt*, including novel SNPs, and confirm the presence of a *dhfr*-164L quadruple mutant in coastal Kenya. Mutations implicated in sulfadoxine-pyrimethamine resistance are at or near fixation in this sample set.

**Conclusions/Significance:**

Sequence-based GWA studies are powerful tools for phenotypic association tests. Using this approach on falciparum parasites from coastal Kenya we identified known and previously unreported genes associated with phenotypic resistance to anti-malarial drugs, and observe in high-resolution haplotype visualizations a possible signature of an inverse selective relationship between CQ and PQ.

## Introduction

*Plasmodium falciparum* malaria is responsible for nearly 600,000 African deaths every year, and in Kenya consumes a fifth of hospitalization resources [1], [2]. Prompt treatment with anti-malarials can prevent mortality, but this efficacy is threatened by the parasite's ability to acquire drug resistance. This highlights the appeal of high-resolution genetic markers and data-sharing for early-warning surveillance [3]. Additionally, the elucidation of genetic loci underlying resistance is important for designing new formulations, and can reveal opposing selective pressures amongst drugs [4].

Drug resistance loci in *P. falciparum* parasites have been discovered using genetic crosses for QTL analysis [5], [6]. A number of recent studies targeted on particular parasite genes in coastal Kenya have described drug activity associations with familiar SNPs in *pfmdr1*, *pfcrt*, and *pfdhfr*, as well as structural associations with quinine (QN) tolerance in *pfnhe* [*7*], [*8*], [*9*]. Population-genetic approaches, such as sequence-based GWAS, provide the advantage of testing for phenotypic associations with novel SNPs while broadly surveying known polymorphisms [10].

This work examines the association between SNPs ascertained from whole-genome sequencing of 35 Kenyan field isolates with the activities of 22 antimalarial drugs (Figure S1, Table S1). The cooperative efforts of the partnerships in the Malaria Genomic Epidemiology Network (MalariaGEN) have created a panel of highly credible SNPs ascertained in the context of 1685 parasites, contributed from 17 countries, and we utilize this community resource here [11].

## Materials and Methods

### Ethics statement

Parasites were isolated from the peripheral blood of participants in two clinical trials on Artekin versus Coartem, conducted in Kilifi between 2005 and 2007. All studies obtained clearance from the Kenya Medical Research Institute (KEMRI) Ethical Review Committee under the protocol numbers SSC 945 and SSC 946.

### Sample collection and processing

Infected blood pellets were cryopreserved using glycerolyte and later adapted to culture as described elsewhere [12]. Pellets were frozen for three months on average before culture adaptation and chemosensitivity testing, and were in continuous culture for approximately two months for these assays before DNA extraction and sequencing (Figure S2). DNA was extracted from adapted field isolates using the QIAamp DNA Blood Mini Kit (Qiagen, UK).

Of the thirty-five isolates used in the final analysis, thirteen were taken from patients admitted to Kilifi District Hospital with severe malaria, and twenty-two from participants in a study comparing Artekin to Coartem [13]. Of these latter twenty-two, twelve were collected at recruitment, and ten were collected 19–84 days later (mean = 48.7days), representing reinfections or recrudescences. Two of the ten follow-up samples are from patients also represented at recruitment in this dataset. We classified both of these cases as reinfections because, based on the number of SNP identities, the recruitment and follow-up parasites were no more similar to one another than to those from other patients.

### Chemosensitivity testing

Details of IC_50_ determination for each parasite isolate have been previously described [9]. For a given assay, duplicate series of 200 ul cultures containing 0.5% parasitemia and 1.5% hematocrit were established in 96-well microtiter plates and exposed to a gradient of drug concentrations. Drug sensitivity was approximated by standard incorporation of tritiated hypoxanthine, added after 24 hours of culture and measured by scintillation 18–20 hours later. The concentration at which 50% growth inhibition was achieved was estimated using nonlinear regression. Chemosensitivity assays were performed two to four different times on each isolate, on separate days, and the median IC_50_ value was used as the phenotype in the final analysis. Median IC_50_ concentrations were determined for each of 22 drugs applied to 59 parasite isolates.

### Sequencing and genotyping

Extracted DNA was contributed to MalariaGEN for whole-genome sequencing and genotyping [11]. Isolates were sequenced with an Illumina Genome Analyzer to a read depth of approximately 98x in genotyped loci, and reads of length 37–76 base pairs were aligned to the 3D7 reference genome as previously described [14]. Genotype calls for each sample were provided by MalariaGEN for more than 400,000 high-quality exonic SNPs in their current catalog of genetic variation. Sequencing data for the parasites used in this study have been deposited in the European Nucleotide Archive, and are publicly available for download (http://www.ebi.ac.uk/ena/). Accession numbers and corresponding phenotype data are listed in Table S6. SNP genomic coordinates and annotations are maintained by MalariaGEN, and the most updated tools for viewing this information can be found at http://www.malariagen.net/data.

Whole-genome sequencing and genotyping was successful for 43 of 59 isolates. Five samples were clear outliers from a dense cluster of the others in initial principal components plots, perhaps due to cross-contamination, and thus were excluded from further analyses. Two samples were removed for having an excessively high proportion of missing SNPs (>60%, vs. less than 10% for most others), and an additional sample was excluded because it was identical at every position to another taken from the same patient one month prior.

### Analysis

All analyses were performed using R and Perl. For each SNP with greater than 9% minor allele frequency (MAF) amongst these 35 samples (N = 6250), an independent hypothesis test was performed to assess whether log_10_(IC_50_) levels differed between the reference (i.e., 3D7-like) and alternate allele groups. This was done separately for each drug. The MAF of 9% was chosen to ensure the minor allele group had at least 3 representative parasites. The SNP-wise hypothesis tests assessed whether the dichotomous fixed effect of genotype (i.e., 3D7 vs. alternate alleles) was equal to zero in a linear model that also contained three surrogate variables to account for population structure. The surrogate variables were calculated from principal components analysis (PCA) performed on a matrix of 35 quality filtered samples and 12802 SNPs, in which each cell was the reference allele frequency. For this PCA, SNPs with no missingness in any sample were included. The first three eigenvectors were projected onto the data, and these variables were modeled as direct, fixed-effects. Although mixed models accommodating within-isolate experimental replicates as random effects improved p-values, we chose to median collapse repeated assays to avoid the possibility of pseudo-replication. Significant SNPs were also tested by Kruskal-Wallis, and residuals assessed for departure from normality by quantile-quantile (QQ) plots and the Shapiro-Wilk test. We used Spearman's rank for pairwise drug correlations and tests. Genome-wide significance was defined as q-value less than 0.05 after correcting for multiple comparisons by estimating the False Discovery Rate [15].

Although we find evidence that substantial within-sample heterozygosity remains after culture adaptation (Figure S3), we decided against modeling complexity of infection (COI) as a continuous genotype after observing that 93% of MAFs fall within 5% of either homozygous extreme (Figure S4). We believe such genetic models are promising for parasites direct from blood, but warrant further investigation in this context, as little is understood about the dynamics of COI as isolates adapt to culture. We therefore decided to adopt a more conservative approach and discard heterozygous observations.

## Results

### GWAS

**Figure 1:**
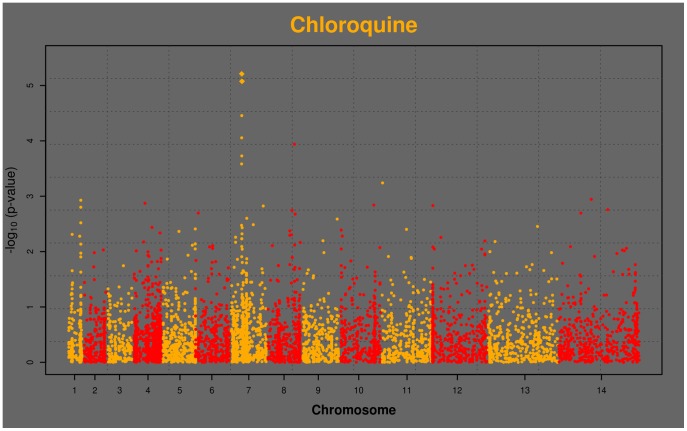
Manhattan plot of genome-wide associations with CQ activities from 35 parasite isolates. Horizontal axis is genome position, and vertical axis is –log_10_(p-value). Chromosomes alternate yellow and red, starting from chromosome 1 on the left. Yellow spire on chromosome 7 is in the region of *pfcrt*.

**Figure 2:**
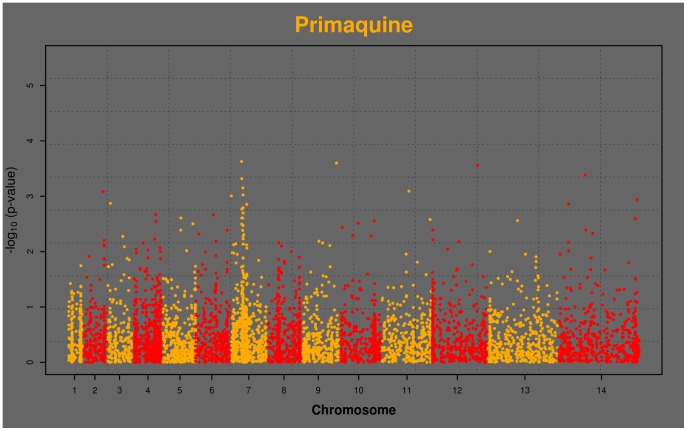
Manhattan plot of genome-wide associations with PQ activities from 35 parasite isolates. Horizontal axis is genome position, and vertical axis is –log_10_(p-value). Chromosomes alternate yellow and red, starting from chromosome 1 on the left. Yellow spire on chromosome 7 is in the region of *pfcrt*.

We tested 6250 SNPs for association with the activities of 22 drugs, and report 11 loci that meet genome-wide significance (Table 1). Two loci were significantly associated with CQ activity, and are within the genes *cg1* and *cg2*, adjacent to *P. falciparum* Chloroquine Resistance Transporter (*pfcrt*). These two genes have frequently been associated with CQ resistance (CQR) in the literature, likely due to LD with *pfcrt* [16], [17]. Two nonsynonymous SNPs in genes on chromosomes 2 and 6 were associated with QN sensitivity, and 5 SNPs with quinazoline activity on chromosomes 5, 9, 11, 13, and 14. We note that although the p-values for several of the QN and quinazoline hits are more significant than those for CQ, the Manhattan plot for CQ exhibits signal from a number of corroborating SNPs in proximity to *pfcrt* that do not reach genome-wide significance (Figure 1). This region is known to have uniquely long-range LD for *falciparum*, a remnant of the selective sweep of CQ resistance through the population [18]. We also notice that primaquine yields similar interesting signal in the *pfcrt* region, though no individual SNP meets genome-wide significance by association alone (Figure 2).

**Table 1:** SNPs achieving significance (q-value<0.05) after correcting p-values for multiple hypothesis tests.

Drug	Gene	p-value	q-value	Gene Definition	AAC^1^
CQ	PF07_0035	6.15E-06	0.031	Cg1 protein	E161**D**
CQ	PF07_0037	0.0000084	0.031	Cg2 protein	L1883**V**
QIN	PFB0870w	6.13E-06	0.023	conserved, unknown function	**E**1771K
QIN	PFF0670w	4.82E-06	0.023	transcription factor, putative	R1034**C**
QuiNazol	PF11_0420	7.7E-07	0.003	conserved, unknown function	**R**1208K
QuiNazol	PF13_0348	9.6E-07	0.003	rhoptry protein	.
QuiNazol	PF14_0726	0.0000134	0.022	conserved, unknown function	**T**207P
QuiNazol	PFE0020c	0.0000067	0.015	rifin	**N**226D
QuiNazol	PFI0495w	3.484E-05	0.046	conserved, unknown function	**L**268F
AQ^2^	PF07_0068	4.04E-06	0.012	cysteine desulfurase, putative	**E**339G
AQ^2^	PF07_0068	4.54E-06	0.012	cysteine desulfurase, putative	**F**361L

1 Amino Acid Change. Synonymous substitutions indicated with a dot. Allele associated with drug tolerance in bold.

2 Meets genome-wide significance without principal components in model (see Results).

Based on the quantile-quantile distribution of associations with the CQ phenotype, we used the first 3 principal components to correct a modestly deflated genome-wide inflation factor (λ = 0.99), and applied this methodology to all drugs [19]. Amodiaquine activities were anticorrelated with the first two projected components (r = −0.33, r = −0.38), dampening signal from two adjacent loci in PF07_0068 that otherwise stood-out with genome-wide significance, so we report these for thoroughness (Figure S5, Table 1). The ranks of these loci remain in the top 10 of AQ associated hits using either approach.

### *pfcrt* haplotypes

Considering previously described *pfcrt* variants only, we observed two haplotypes representing 28 samples. For this particular analysis we excluded samples that were ambiguous due to missing genotype data or heterozygosity. Visualization of the haplotypes in this region highlights that this gene is difficult to assay with short reads, and explains why tagging SNPs of K76T yielded the strongest GWAS signal. At amino acid positions 72, 74–76, and 271, twenty isolates have residues CMNKQ, and 8 carry CIETE (Table S4). We also detected non-synonymous variants at two other loci (positions 24 and 124), that partitioned the 20 CMNKQ parasites into 3 haplotypes: 17 with DR at these positions, one with DQ, and two with amino acids YR (Table S4).

### pfdhfr, pfdhps, and pfmdr1

Resistance to the antifolates pyrimethamine and sulfadoxine is attributed, respectively, to point mutations in *dhfr* and *dhps*, but we found no significant associations with loci in either gene [20]. This was expected, as we did not test the activity of sulfadoxine, and the pyrimethamine resistance-conferring *dhfr* S108N mutation is at fixation in our samples (Figure S6, Table S2). Positions 51I and 59R in *dhfr* are nearly fixed as well, and we detected the presence of one quadruple (I164L) mutant in a mixed infection, corroborating previous reports of the emergence of this allele in Madagascar and coastal Kenya [7], . Excluding mixed infections, we observed no occurrences in *dhps* of 437A-540E double mutants, but every parasite carried one or the other (Figure S7).

Similarly, we discovered no signals of association in *pfmdr1*. A previous study found an association of *pfmdr1* position 86 mutants with lumafantrine (LUM) susceptibility in coastal Kenya, however this SNP failed to meet our quality thresholds, as did position 1246 [8]. Further, we observed little variation in this gene in SNPs that might otherwise have tagged position 86, or other commonly implicated loci (Figure S8). A larger sample size would be necessary to detect very low frequency variants in this gene.

### pfnhe

Previous reports have associated structural variants in the sodium/hydrogen exchanger gene (*pfnhe*) with quinine tolerance *in vitro* [9], [22]. These structural variants in microsatellite ms4760 of *pfnhe* may be important markers for surveillance, and more work is needed to describe the natural variation in this gene [23]. While the analysis of structural variation is beyond the scope of this particular output, we do report 15 nonsynonymous SNPs in *pfnhe* (Table S3). N894K has been previously described and appears in 4 isolates. The most common variant was carried in 6 isolates (D209Y).

### Drug correlations

**Figure 3:**
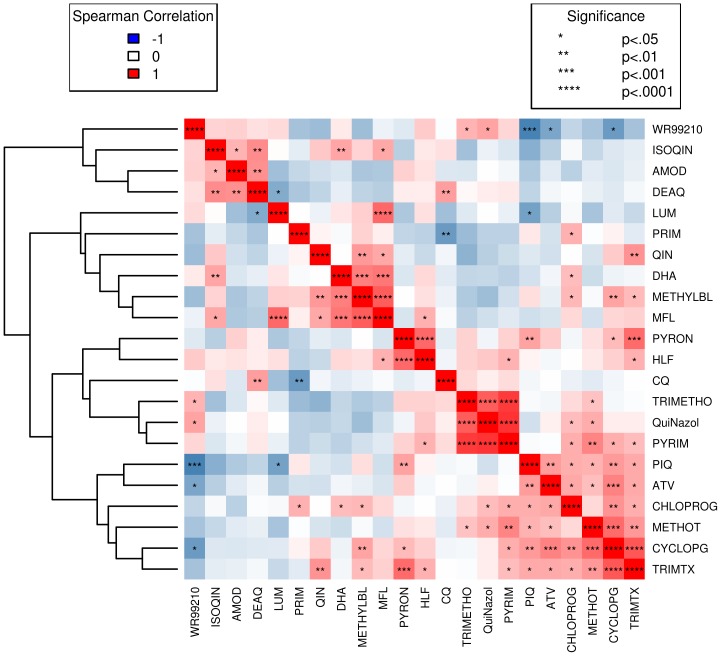
Cluster plot of drug correlations. Red to blue indicates the degree of positive to negative correlation. Significance levels of spearman rank tests are indicated with stars in each box (see legend).

Drugs with correlated activities may indicate related mechanisms of action, and perhaps more importantly, those with negative correlations might reveal synergistic partners for co-deployment or rotation strategies [24]. Several drugs, including lumafantrine, have been reported to select for parasites with inverse susceptibilities to CQ, and we find evidence of this as well (Figure 3) [25]. CQ activity is significantly correlated with desethylamodiaquine (DEAQ, r = 0.49, p = 0.006) and anticorrelated with PQ (r = −0.48, p = 0.008). Related to this, *pfcrt* haplotypes associated with CQ resistance sort inversely to PQ activity, and yield association signal in the same region (see Discussion). Interestingly, the *dhfr*-targeting drug, WR99210, is negatively correlated with many of the other antifolates. Exceptions to this include trimethoprim, quinazoline, and pyrimethamine, which themselves form a tightly related cluster. Piperaquine activity is more highly correlated with the antifolates than with the aminoquinolines, with the exception of pyronaradine. Piperaquine and other bisquinolines have demonstrated effectiveness against CQ resistant parasites *in vitro*, and another study in coastal Kenya found no association of *pfcrt* with activity for this drug [8], [26].

## Discussion

We detect expected signals of association with CQ activity in the *pfcrt* region with these 35 samples. CQ was a highly effective and cheap drug in Kenya for decades before the emergence of resistance in the mid-1980s [27]. National policy shifted from CQ to the antifolate, SP, in 1998, to which resistance also emerged in a short time [28]. Resistance to CQ remains above 60% in Kenya, and prevalence of the important chloroquine resistance (CQR) conferring K76T mutation was measured at 63% in the coastal region in 2006 [29], [30]. A hallmark of selective sweeps, like that of CQR in Kenya, is uncharacteristically long haplotypes; i.e., segregating stretches of DNA carrying the resistance-conferring allele that have yet to be broken down by recombination. One of the significant CQ associated SNPs we find within *pfcrt*, Q271E, is in complete LD with K76T for these samples—consistent with a report 4000 miles away in Senegal [31]. This level of LD might prove useful for imputation in similar populations of the important K76T variant, which is in a region we find relatively difficult to access with short-read sequencing. Indeed, outside of Papua New Guinea and South America, we find 99.8% agreement (1041/1043) between these two positions in homozygous MalariaGEN samples. Thirty-four percent of the Kenyan isolates used in this study carry the K76T substitution (46% if missing calls are inferred by Q271E).

We also report potentially novel associations for quinine, quinazoline, and amodiaquine. AQ tolerance is commonly associated with *pfcrt*, however this drug remains effective against some CQ resistant parasites—i.e., *pfcrt* alone does not encapsulate resistance [32], [33]. The CIET haplotype observed in this study is not sufficient in isolation for conferring AQ resistance, and we do not detect significant signal for this drug in *pfcrt* [34], [35], [36]. We report two SNPs in a putative cysteine desulfurase gene (PF07_0068) that are significantly associated with AQ activity (Table 1). This gene is more than 300Kb from *pfcrt*, thus not likely tagging the CQR haplotype. 4-aminoquinolinines like CQ and AQ are thought to act by accumulating in the parasite digestive vacuole (DV) and preventing the crystallization of heme dimers into hemozoin [37]. The elevated concentration of toxic heme within the DV leads to increased efflux into the cytosol in a dose-dependent manner, resulting in an oxidative challenge to the parasite and membrane damage. Free heme should be detoxified by glutathione in the cytosol, but both CQ and AQ directly compete with this activity [38]. One might speculate whether cysteine desulfurase affects this interaction, or is more broadly involved in parasite pathways related to alleviating increased oxidative stress, for example the thioredoxin or glutathione redox systems. In plants, cysteine desulfurase has been postulated to modify the catalytic properties of glutathione by changing cysteine content [39].

A decade after CQ withdrawal in Malawi, the proportion of circulating CQR parasites in the population has receded to nearly undetectable levels [40]. The velocity of this particular shift appears to be somewhat unique, nonetheless CQR in Kenya has also been on the decline since CQ withdrawal in 1999 [29]. The haplotypes and patterns of LD support that this event in Malawi was due to an expansion of the existing CQ susceptible (CQS) parasite population, rather than a sweep or reversion, and our data are consistent with this model as well [41], [42]. All parasites with the resistant *pfcrt*-76T allele are represented by a single haplotype across 7 positions, in contrast to the susceptible forms which are comprised of several haplotypes. This is consistent with the hypothesis that, relative to the homogenous CQR parasites originating from a selective sweep, a diverse pool of susceptible parasites has been maintained and serves as a reservoir of expansion in the absence of drug pressure. This stands-out visually when a second haplotype in *cg1*, found 2kb downstream, is juxtaposed with *pfcrt* (Figure S9). Although our inferences are limited due to small sample size, it would appear that CQS diversity was not completely extinguished under decades of drug pressure, indicated by the higher relative polymorphism in the parasites that are both most susceptible to CQ, and lack the 76T allele.

**Figure 4:**
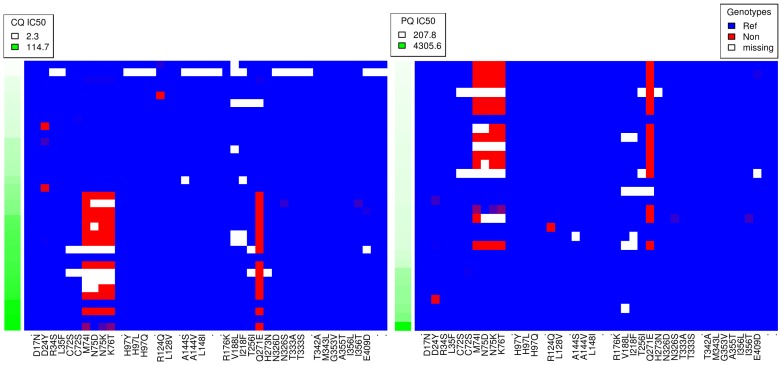
Haplotype plot for *pfcrt* (MAL7P1.27), sorted by CQ and PQ activities. Left panel is sorted top to bottom by increasing CQ IC_50_, and the right panel is sorted by PQ IC_50_. Each row represents a sample, and each column a potential SNP. Drug activity is shown as increasing green intensity in the far left column of each plot. Blue cells indicate positions matching the reference genome, and red the alternate allele. Mixed infections are represented by blending of red and blue, proportional to the within-sample allele frequencies. White cells indicate missing data. Nonsynonymous SNPs are labeled with the amino acid substitution along the bottom, and with a dot if synonymous.

Like verapamil (VP), PQ has been shown to reverse CQ resistance in a dose-dependent manner, and there is evidence supporting direct inhibition of *pfcrt* as the underlying mechanism [43], [44], [45]. It is therefore intriguing that we observe negatively correlated PQ and CQ activities, and correspondingly inverse *pfcrt* haplotype plots when sorted by drug activities (Figure 4, Table S5). Both PQ and CQ phenotypes yield convincing GWAS signal in the *pfcrt* region as well. Of the top 17 SNPs (by p-value) for these two drugs, 3 SNPs overlap identically in the CRT region, and another half-dozen are in the same vicinity, all of which have consistently inverse trends. It is tempting to speculate that in addition to PQ interacting with CRT mutants to reverse resistance directly, CQ might, separately, select for parasites that are more susceptible to PQ. If confirmed, the relevance of this would depend on whether the biochemical target of the high concentrations required for shizontocidal activity here is the same mechanism conventionally affected by lower concentrations in other stages. Primaquine's precise mechanism of action is unknown [46]. We cannot make statements about whether primaquine, in reverse, would select for CQ sensitive parasites, as it is unlikely that our isolates were exposed to natural primaquine pressure. Primaquine is primarily used for clearing *P. vivax* and *P. ovale* hypnozoites, and although it also has activity against gametocytes, this community benefit is counter-balanced by the risk of hemolysis to G6PD deficient individuals [47]. Evidence of selective interactions as we report here would be salient in such drug policy decisions. A similar study in Senegal reported a highly significant signal of selection for PQ sensitivity in the *pfcrt* region, and those authors attribute this to PQ anticorrelation with CQ [48]. With regard to selection, such relationships are not unprecedented—e.g., inverse pressures on *pfcrt* between CQ and LUM have been described in Tanzania and Kenya previously [8], [25]. Lumafantrine is the partner drug in the artemisinin-based combination therapy (ACT), Coartem, which has been the first-line treatment for uncomplicated malaria in Kenya since 2006. Although not as strong as with PQ, we similarly observe a modest “flip” in the ordering of haplotypes when CQ is compared to LUM (Figure S10). We caution that with only 35 parasites and a sample limited in time and geography, replicate studies and experiments are needed to confirm these observations.

If adequately powered, null results from GWA studies of drug sensitivities are informative about which therapies might be most effectively deployed in the region of inference. Consistent with overlapping studies in the Kilifi region, we find no association of pyronaradine, methylene blue, piperaquine, or DHA activities with *pfcrt, pfmdr1*, or any other loci [8], [49]. The combination therapy of piperaquine and DHA (Artekin) might therefore be currently effective in this population, even with some degree of CQR prevalence. We reinforce that with our limited sample size, interpretations of null associations must be heavily tempered; nonetheless, this study contributes precedent for planning future genome-wide association and surveillance studies.

In summary, we confirm the expected signals of association with chloroquine, and report novel loci related to the activities of AQ, QN, and quinazoline. The high resolution provided by sequence-based genotypes also revealed new polymorphisms in current candidates, and provided for haplotype visualizations that highlight relationships otherwise easily overlooked. Notably, these relationships are consistent with other reports, and if validated would be important for ethics and policy decisions involving PQ. Coastal Kenya has experienced a marked decline in transmission intensity over the past decade, and it is important to monitor the resulting dynamic immuno-epidemiology in parallel with the changing parasite population [50]. These developments, and the repeated emergence of drug resistance in Kenya, underscore the urgency for well-powered, sequence-based, genome-wide approaches to genetic association and surveillance of *Plasmodium falciparum*.

## Supporting Information

Figure S1 Histograms of log_10_(IC_50_) values for 22 drugs.
(TIFF)

Figure S2 Workflow of experiment and analysis.
(TIF)

Figure S3 Heatmap depicting the level of heterozygosity in the sample set. SNPs (rows) are ordered by chromosome position. Samples (columns) are ordered by hierarchical clustering of Euclidean distances, based on the indicator variable 0 = heterozygous, 1 = homozygous.
(TIFF)

Figure S4 Histogram of within-sample allele frequencies. Red indicates the 7% of the data falling in the allele frequency range 0.05 to 0.95.
(TIFF)

Figure S5 Manhattan plots for each of 22 drugs tested for association with 6250 SNPs in 35 parasite isolates. Chromosomes are numbered on the horizontal axis. Points alternate yellow and red based on chromosome. Vertical axis depicts negative log_10_(IC_50_) and all plots have the same max of 7.
(TIFF)

Figure S6 Haplotype plot for *pfdhfr* (PFD0830w). Each row represents a sample, and each column a potential SNP. Samples are sorted by pyrimethamine IC_50_, indicated by the green bar on the far left. Blue cells indicate positions matching the reference genome, and red the alternate allele. Mixed infections are represented by blending of red and blue, proportional to the within-sample allele frequencies. White cells indicate missing data. Nonsynonymous SNPs are labeled with the amino acid substitution along the bottom, and with a dot if synonymous.
(TIFF)

Figure S7 Haplotype plot for *pfdhps* (PF08_0095). Each row represents a sample, and each column a potential SNP. Samples are sorted by pyrimethamine IC_50_, indicated by the green bar on the far left. Blue cells indicate positions matching the reference genome, and red the alternate allele. Mixed infections are represented by blending of red and blue, proportional to the within-sample allele frequencies. White cells indicate missing data. Nonsynonymous SNPs are labeled with the amino acid substitution along the bottom, and with a dot if synonymous.
(TIFF)

Figure S8 Haplotype plot for *pfmdr1* (PFE1150w). Each row represents a sample, and each column a potential SNP. Samples are sorted by chloroquine IC_50_, indicated by the green bar on the far left. Blue cells indicate positions matching the reference genome, and red the alternate allele. Mixed infections are represented by blending of red and blue, proportional to the within-sample allele frequencies. White cells indicate missing data. Nonsynonymous SNPs are labeled with the amino acid substitution along the bottom, and with a dot if synonymous.
(TIFF)

Figure S9 Haplotype plot for *pfcrt* (MAL7P1.27) and *cg1* (PF07_0035) combined. Each row represents a sample, and each column a potential SNP. Samples are sorted by chloroquine IC_50_, indicated by the green bar on the far left. Blue cells indicate positions matching the reference genome, and red the alternate allele. Mixed infections are represented by blending of red and blue, proportional to the within-sample allele frequencies. White cells indicate missing data. SNPs in *pfcrt* are indicated with a “*” along the bottom, and those in *cg1* with the “|” symbol. More diversity is apparent in the top rows—i.e., those parasites that are most susceptible to CQ, and lack the 76T allele.
(TIFF)

Figure S10 Haplotype plot for *pfcrt* (MAL7P1.27). Left panel is sorted top to bottom by CQ IC_50_, and the right panel is sorted by LUM IC_50_. Each row represents a sample, and each column a potential SNP. Drug activity is shown as increasing green intensity in the far left column of each plot. Blue cells indicate positions matching the reference genome, and red the alternate allele. Mixed infections are represented by blending of red and blue, proportional to the within-sample allele frequencies. White cells indicate missing data. Nonsynonymous SNPs are labeled with the amino acid substitution along the bottom, and with a dot if synonymous.
(TIF)

Table S1 List of drugs and abbreviations used in this study.
(DOCX)

Table S2 Amino acid haplotypes of hallmark variants in *pfdhps* and *pfdhfr*. Column ‘N’ is the number of samples in this study represented by that haplotype.
(DOCX)

Table S3 Variants detected in *pfnhe*. Column ‘N’ is the number of samples in this study carrying that allele.
(DOCX)

Table S4 Amino acid haplotypes of variants in *pfcrt*. Column ‘N’ is the number of samples in this study represented by that haplotype.
(DOCX)

Table S5 Pairwise drug correlations.
(DOCX)

Table S6 European Nucleotide Archive accession numbers and corresponding phenotypic data for the 35 samples used in this study. Drug abbreviations and concentration units are described in table S1.
(DOCX)
