# Supplementary figures and images for: A Genome Wide Association Study of Plasmodium falciparum Susceptibility to 22 Antimalarial Drugs in Kenya
Source: PLoS One. 2014 May 8;9(5):e96486. doi: 10.1371/journal.pone.0096486 (PMC4014544; doi:10.1371/journal.pone.0096486)

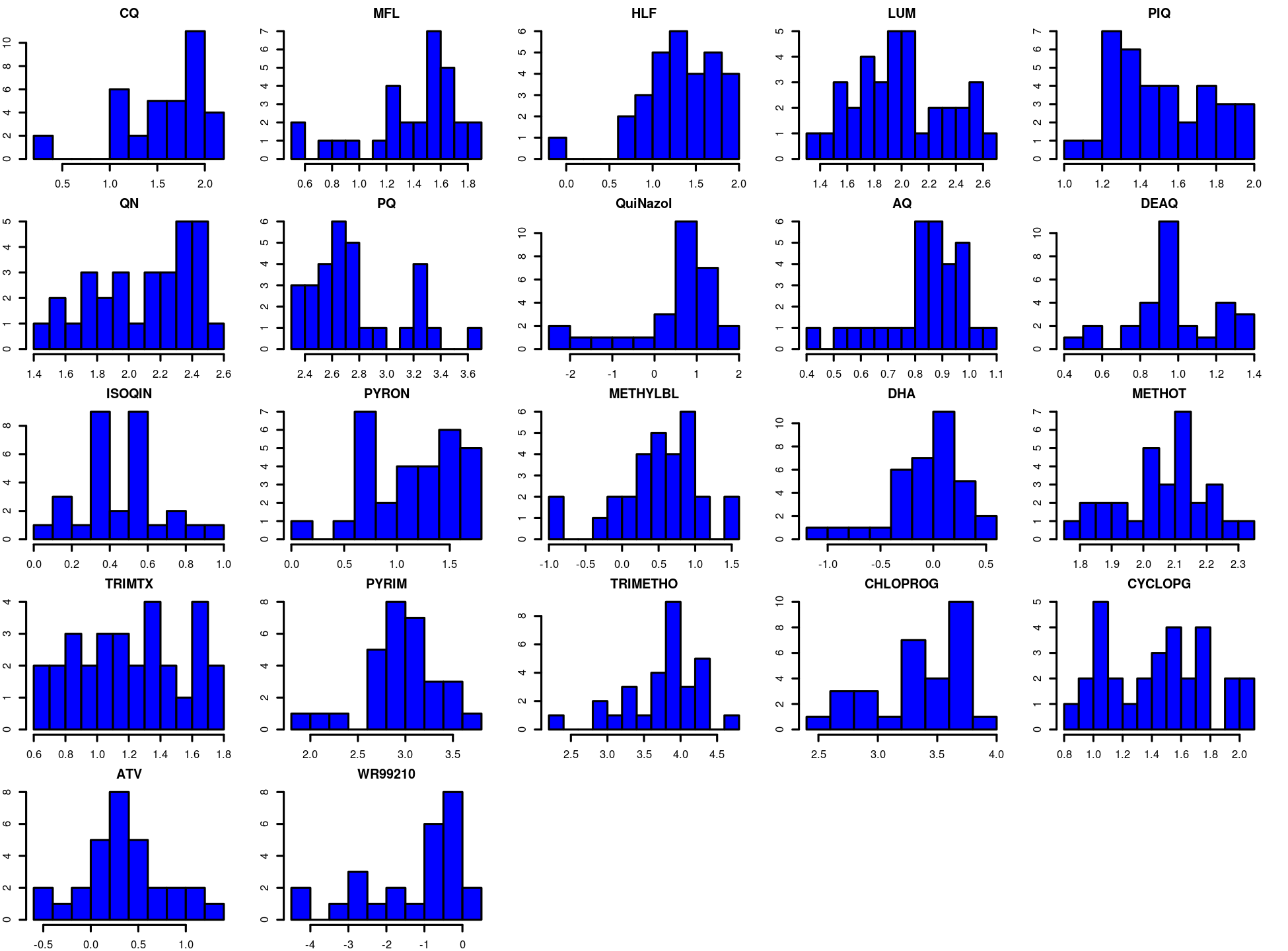

Supplement: Figure S1 — Histograms of log10(IC50) values for 22 drugs. (TIFF) [file pone.0096486.s001.tiff]

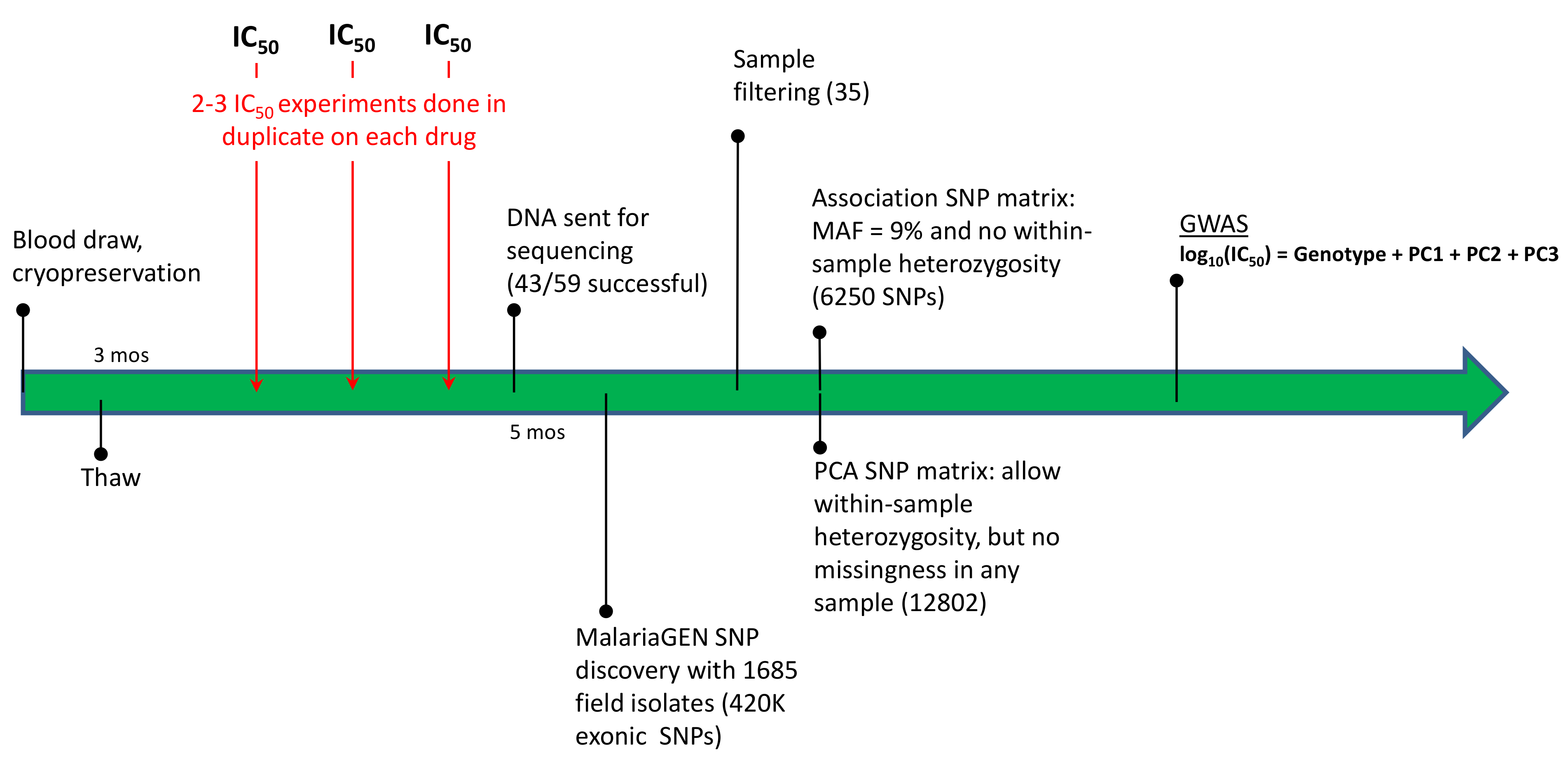

Supplement: Figure S2 — Workflow of experiment and analysis. (TIF) [file pone.0096486.s002.tif]

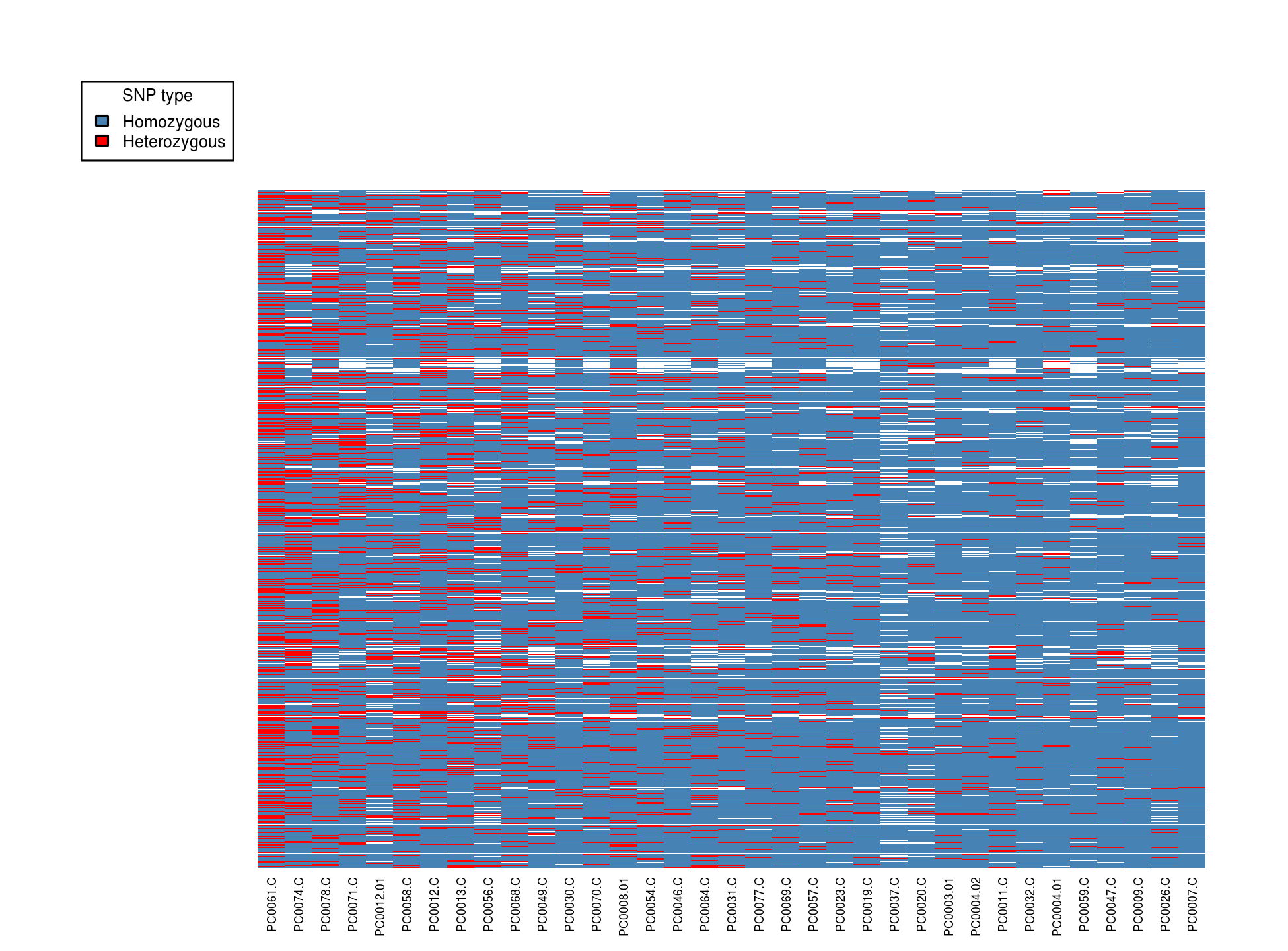

Supplement: Figure S3 — Heatmap depicting the level of heterozygosity in the sample set. SNPs (rows) are ordered by chromosome position. Samples (columns) are ordered by hierarchical clustering of Euclidean distances, based on the indicator variable 0 = heterozygous, 1 = homozygous. (TIFF) [file pone.0096486.s003.tiff]

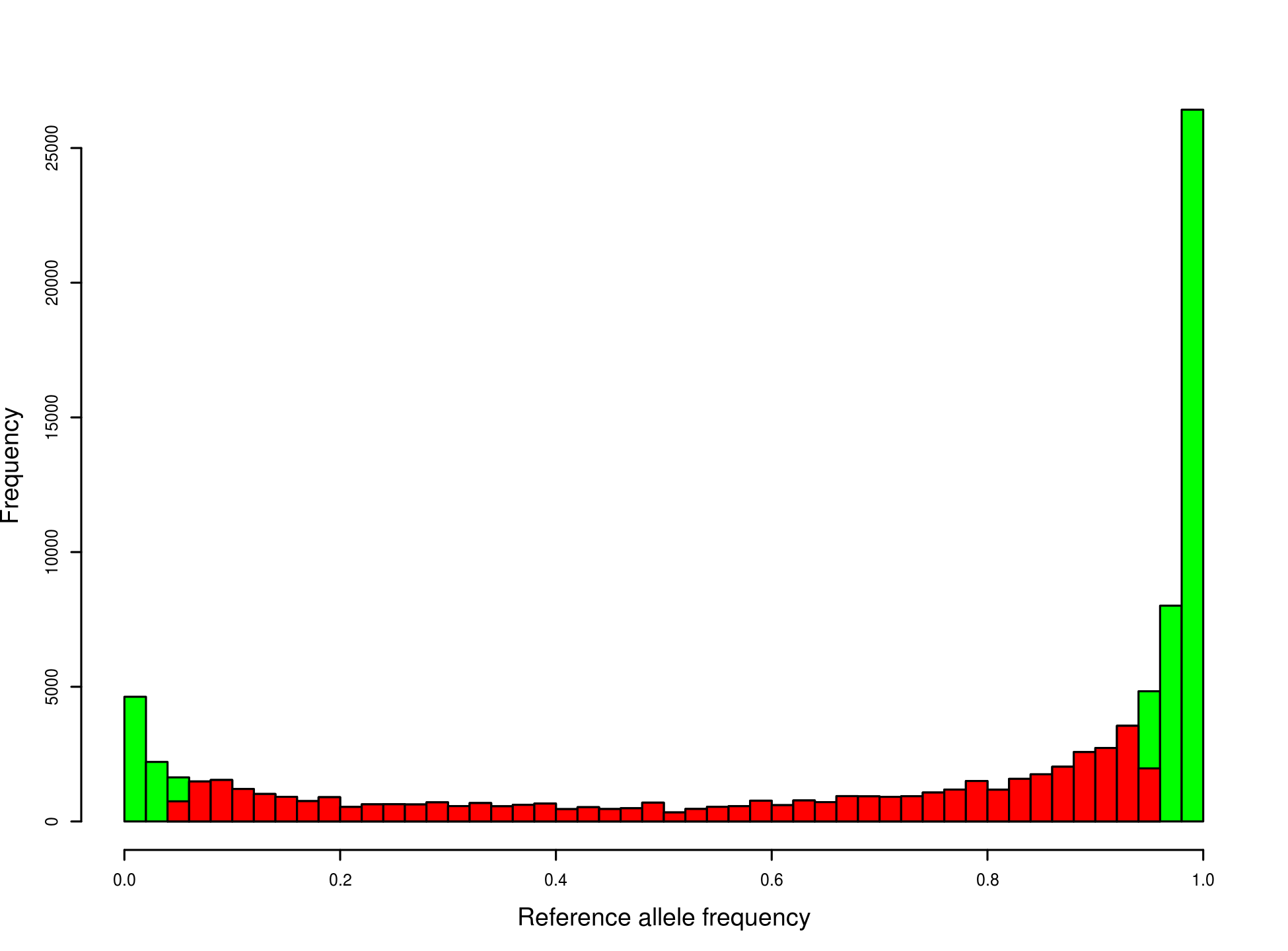

Supplement: Figure S4 — Histogram of within-sample allele frequencies. Red indicates the 7% of the data falling in the allele frequency range 0.05 to 0.95. (TIFF) [file pone.0096486.s004.tiff]

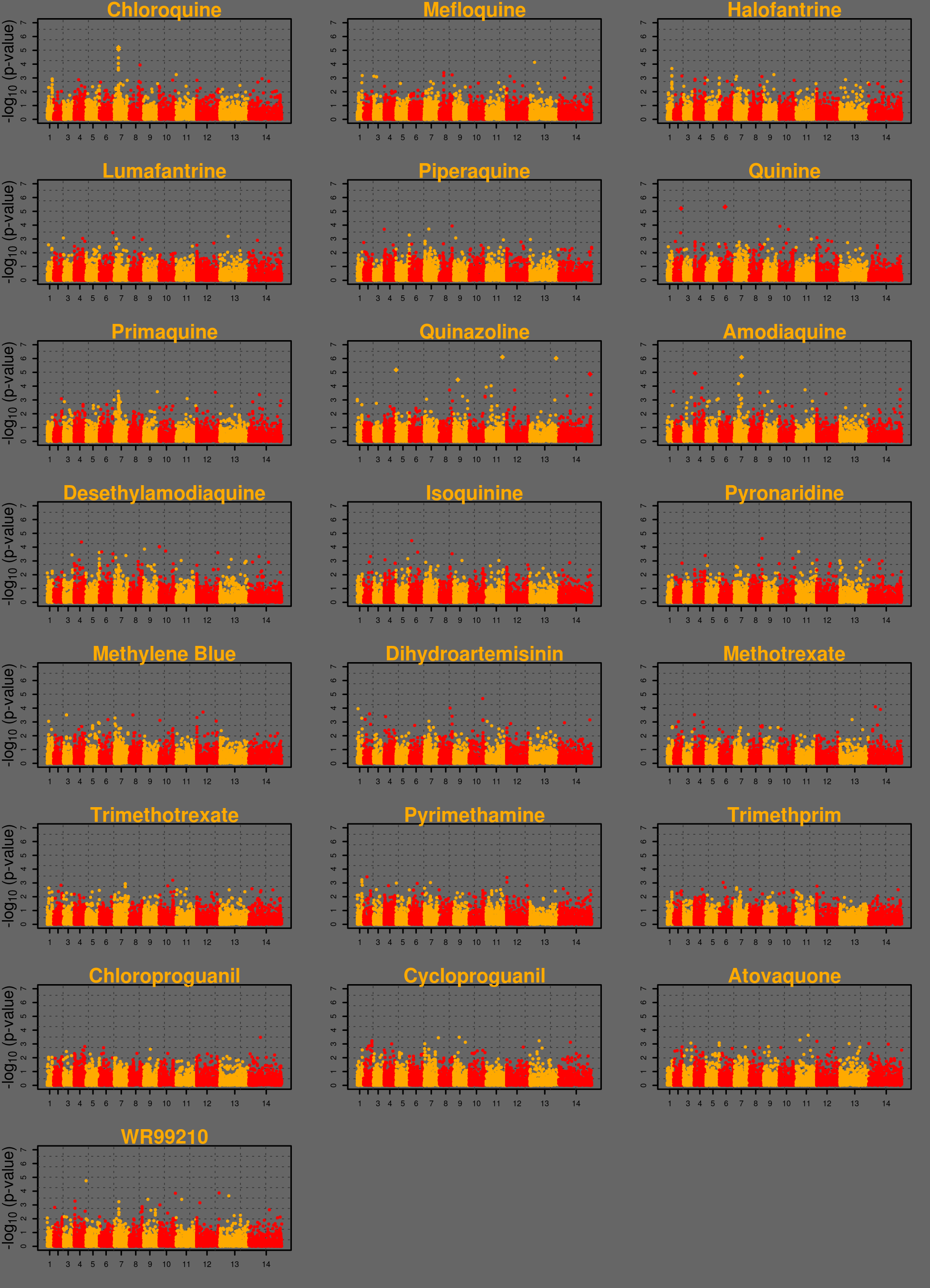

Supplement: Figure S5 — Manhattan plots for each of 22 drugs tested for association with 6250 SNPs in 35 parasite isolates. Chromosomes are numbered on the horizontal axis. Points alternate yellow and red based on chromosome. Vertical axis depicts negative log10(IC50) and all plots have the same max of 7. (TIFF) [file pone.0096486.s005.tiff]

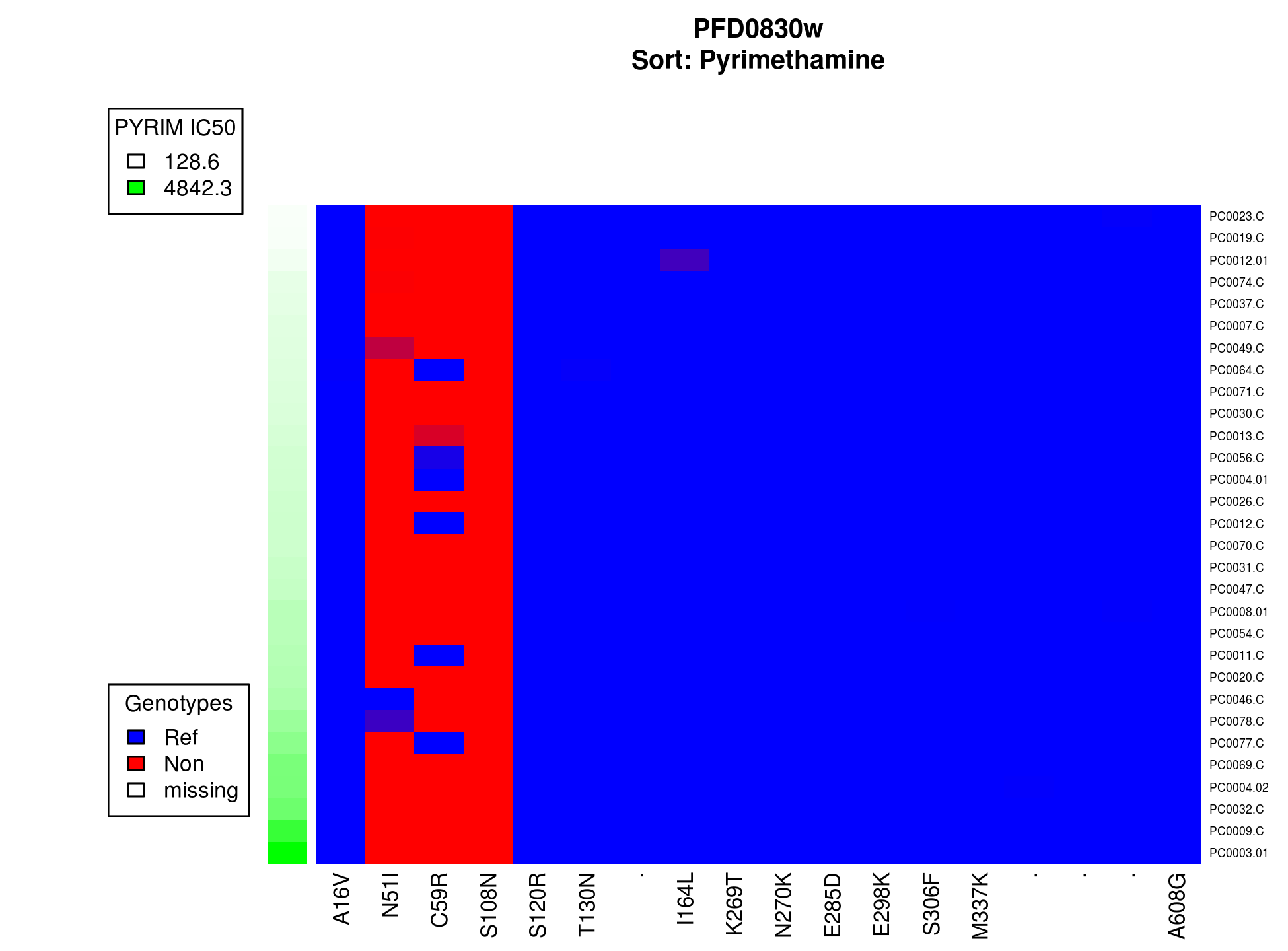

Supplement: Figure S6 — Haplotype plot for pfdhfr (PFD0830w). Each row represents a sample, and each column a potential SNP. Samples are sorted by pyrimethamine IC50, indicated by the green bar on the far left. Blue cells indicate positions matching the reference genome, and red the alternate allele. Mixed infections are represented by blending of red and blue, proportional to the within-sample allele frequencies. White cells indicate missing data. Nonsynonymous SNPs are labeled with the amino acid substitution along the bottom, and with a dot if synonymous. (TIFF) [file pone.0096486.s006.tiff]

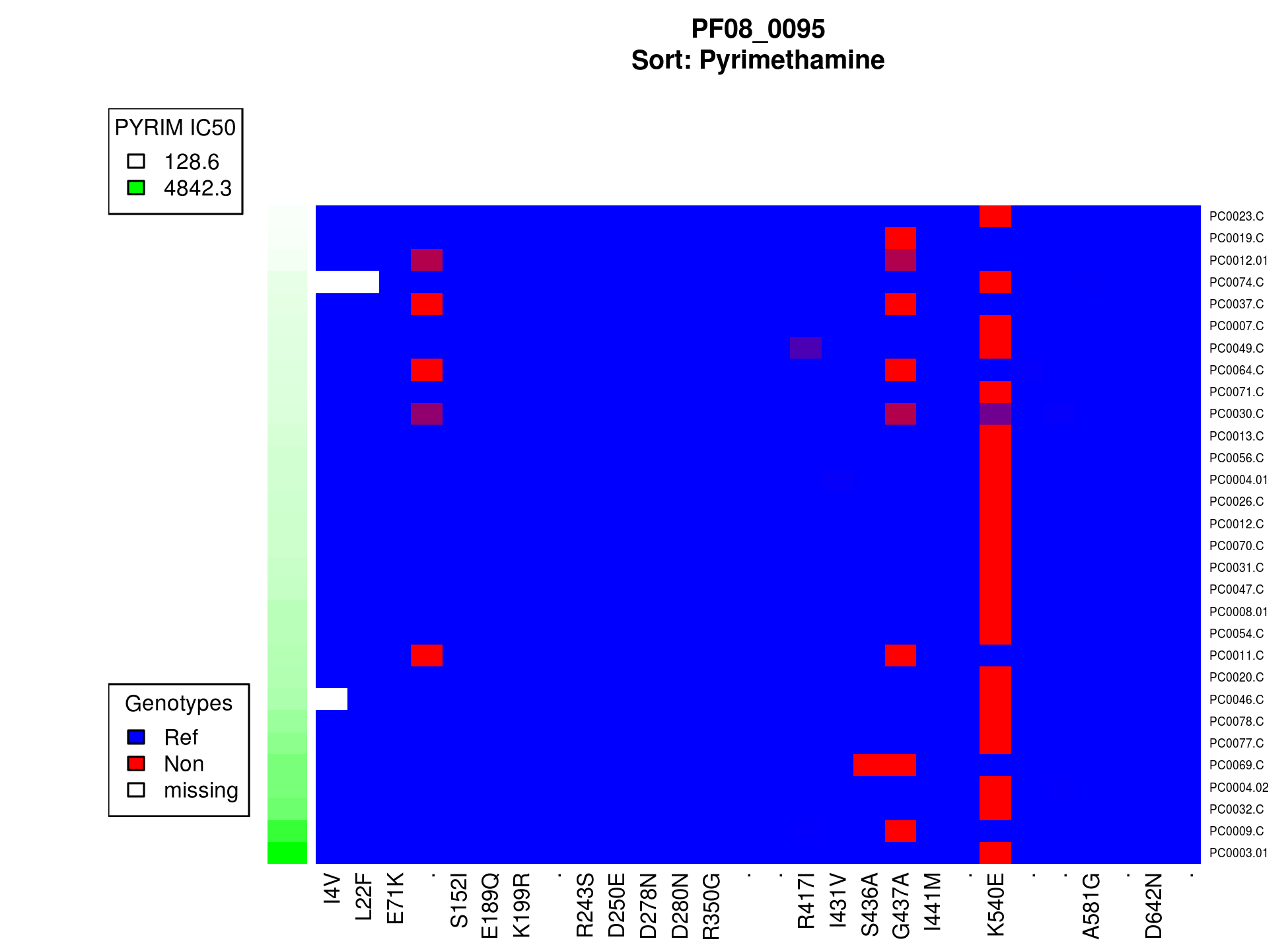

Supplement: Figure S7 — Haplotype plot for pfdhps (PF08_0095). Each row represents a sample, and each column a potential SNP. Samples are sorted by pyrimethamine IC50, indicated by the green bar on the far left. Blue cells indicate positions matching the reference genome, and red the alternate allele. Mixed infections are represented by blending of red and blue, proportional to the within-sample allele frequencies. White cells indicate missing data. Nonsynonymous SNPs are labeled with the amino acid substitution along the bottom, and with a dot if synonymous. (TIFF) [file pone.0096486.s007.tiff]

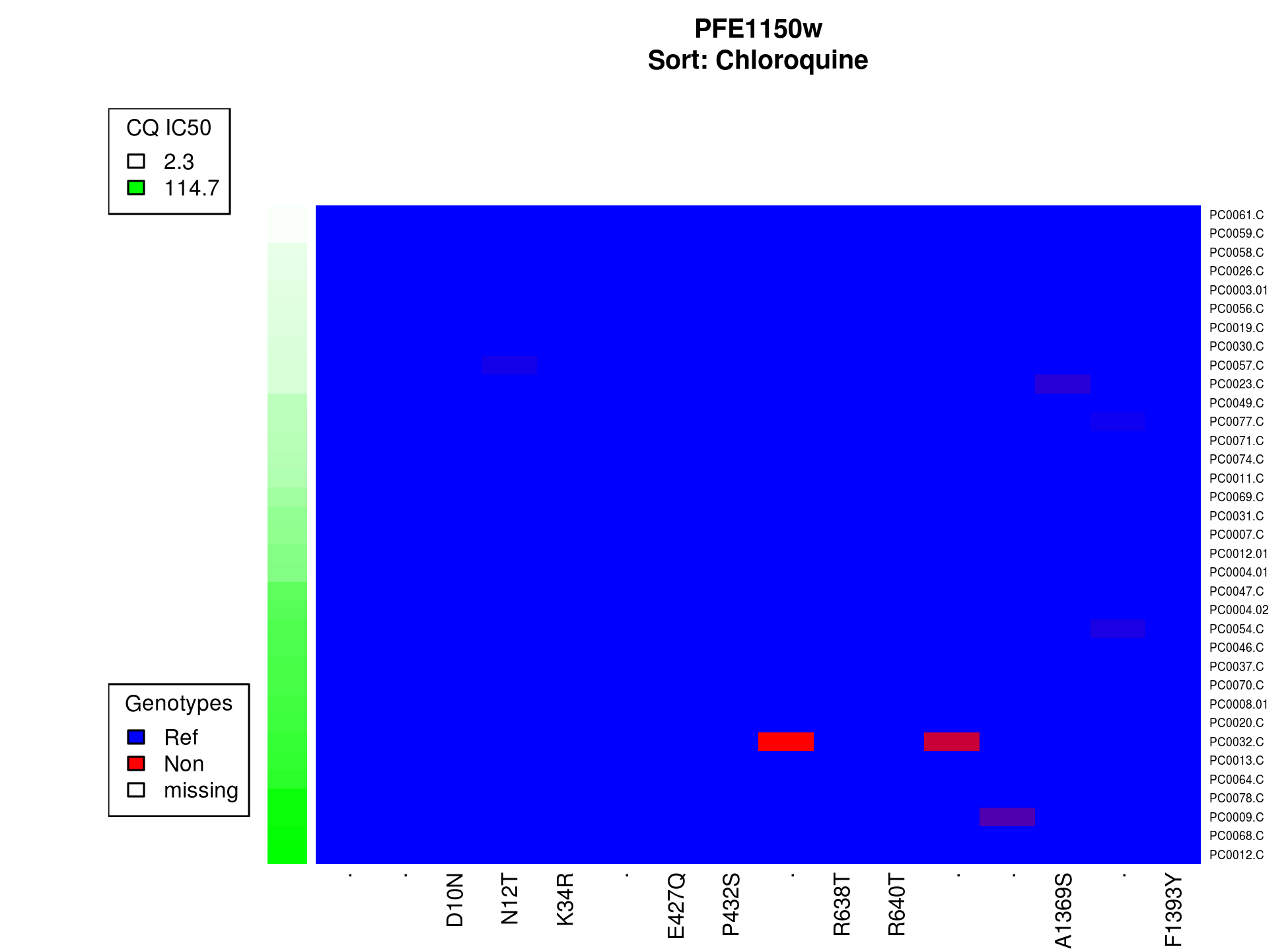

Supplement: Figure S8 — Haplotype plot for pfmdr1 (PFE1150w). Each row represents a sample, and each column a potential SNP. Samples are sorted by chloroquine IC50, indicated by the green bar on the far left. Blue cells indicate positions matching the reference genome, and red the alternate allele. Mixed infections are represented by blending of red and blue, proportional to the within-sample allele frequencies. White cells indicate missing data. Nonsynonymous SNPs are labeled with the amino acid substitution along the bottom, and with a dot if synonymous. (TIFF) [file pone.0096486.s008.tiff]

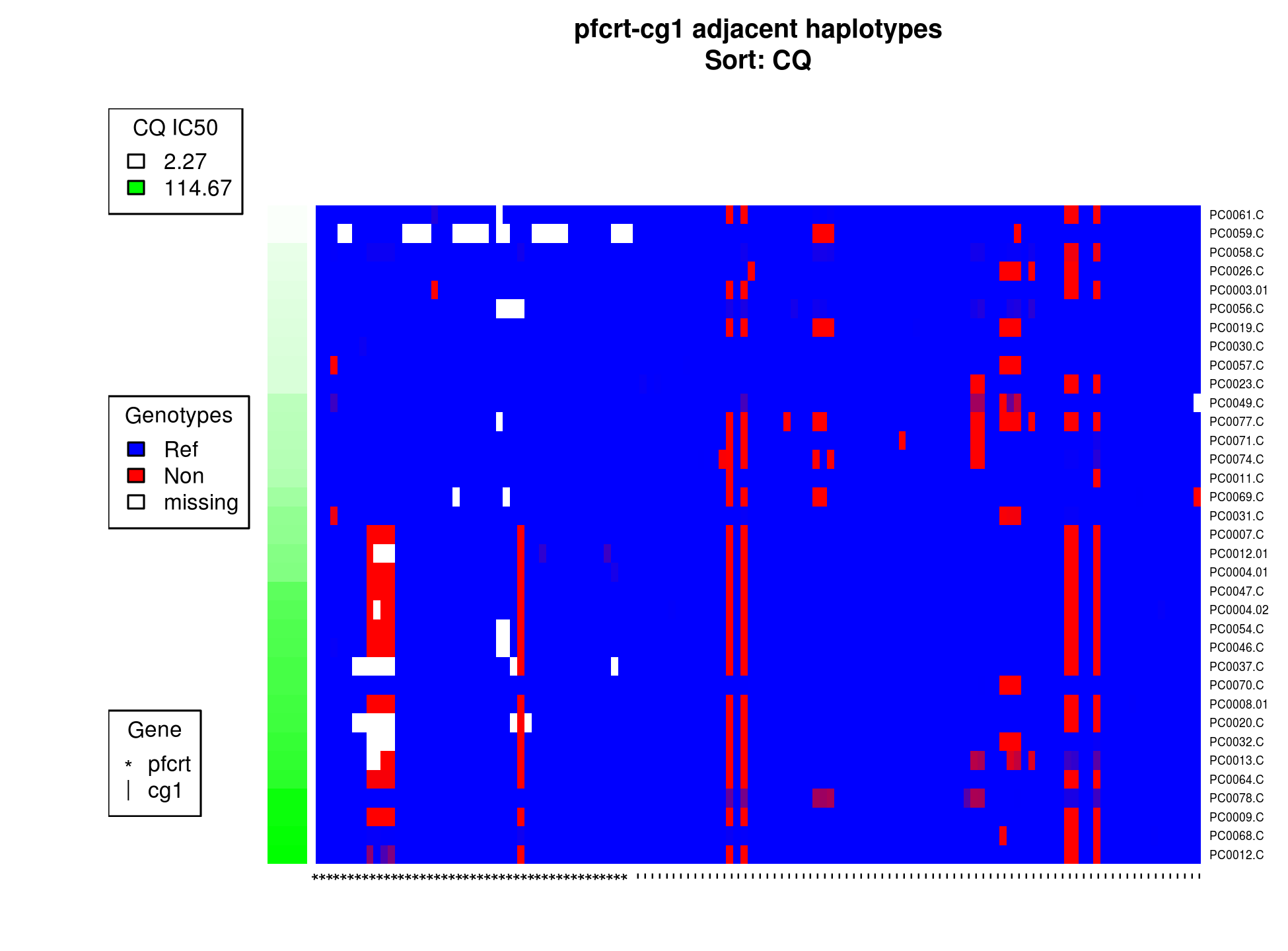

Supplement: Figure S9 — Haplotype plot for pfcrt (MAL7P1.27) and cg1 (PF07_0035) combined. Each row represents a sample, and each column a potential SNP. Samples are sorted by chloroquine IC50, indicated by the green bar on the far left. Blue cells indicate positions matching the reference genome, and red the alternate allele. Mixed infections are represented by blending of red and blue, proportional to the within-sample allele frequencies. White cells indicate missing data. SNPs in pfcrt are indicated with a “*” along the bottom, and those in cg1 with the “|” symbol. More diversity is apparent in the top rows—i.e., those parasites that are most susceptible to CQ, and lack the 76T allele. (TIFF) [file pone.0096486.s009.tiff]

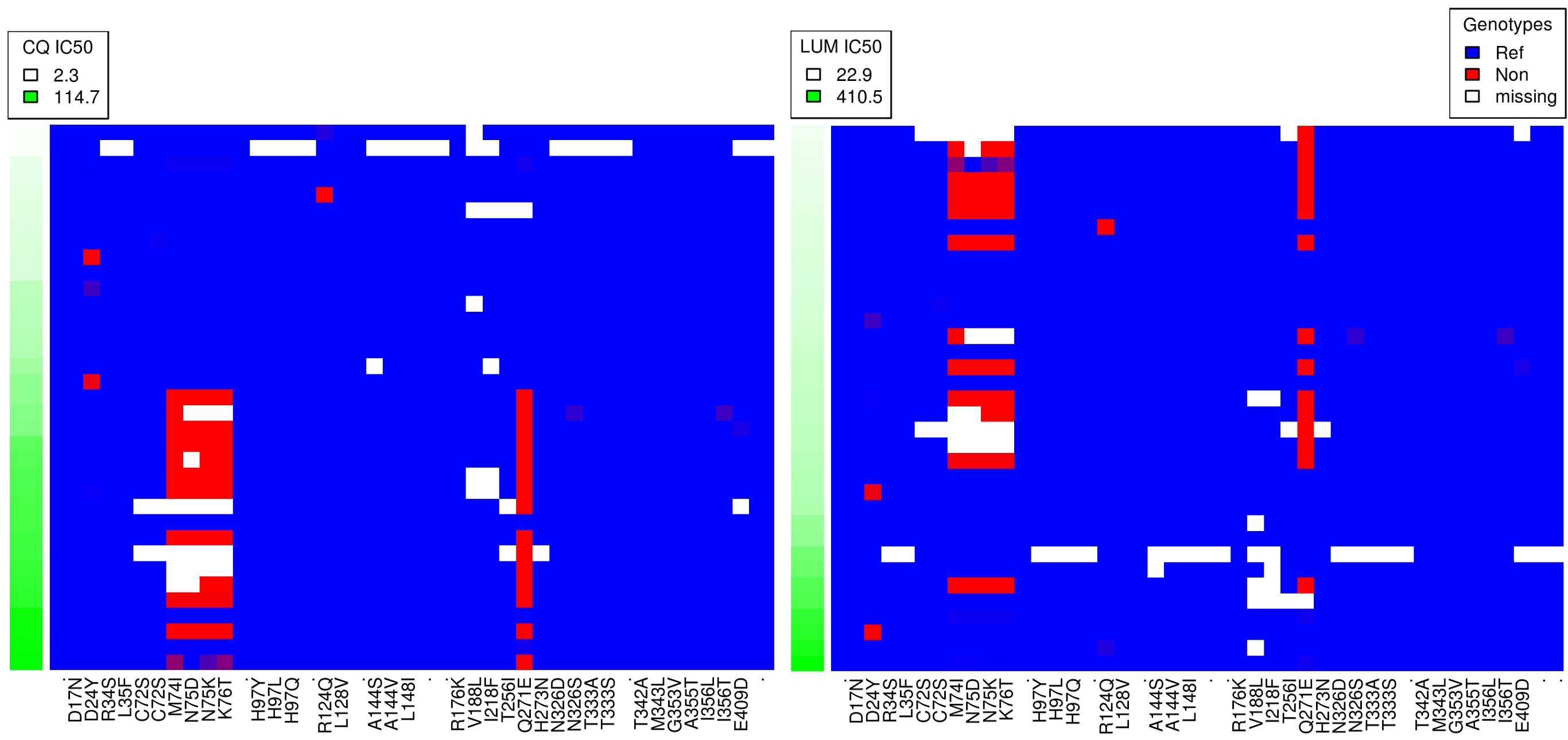

Supplement: Figure S10 — Haplotype plot for pfcrt (MAL7P1.27). Left panel is sorted top to bottom by CQ IC50, and the right panel is sorted by LUM IC50. Each row represents a sample, and each column a potential SNP. Drug activity is shown as increasing green intensity in the far left column of each plot. Blue cells indicate positions matching the reference genome, and red the alternate allele. Mixed infections are represented by blending of red and blue, proportional to the within-sample allele frequencies. White cells indicate missing data. Nonsynonymous SNPs are labeled with the amino acid substitution along the bottom, and with a dot if synonymous. (TIF) [file pone.0096486.s010.tif]
