# Supplementary material for: A Genome Wide Association Study of Plasmodium falciparum Susceptibility to 22 Antimalarial Drugs in Kenya
Source: PLoS One. 2014 May 8;9(5):e96486. doi: 10.1371/journal.pone.0096486 (PMC4014544; doi:10.1371/journal.pone.0096486)
Supplement: Table S1 — List of drugs and abbreviations used in this study. (DOCX) [file pone.0096486.s011.docx]

| **Drug** | **Abbreviation** | **Units** |
| --- | --- | --- |
| Amodiaquine | AMOD, AQ | nanomol |
| Atovaquone | ATV | nanomol |
| Chloroproguanil | CHLOPROG | nanomol |
| Chloroquine | CQ | nanomol |
| Cycloproguanil | CYCLOPG | nanomol |
| Desethylamodiaquine | DEAQ | nanomol |
| Dihydroartemisinin | DHA | nanomol |
| Halofantrine | HLF | nanomol |
| Isoquinine | ISOQIN | ng/ml |
| Lumafantrine | LUM | nanomol |
| Methotrexate | METHOT | nanomol |
| Methylene Blue | METHYLBL | nanomol |
| Mefloquine | MFL | nanomol |
| Piperaquine | PIQ | nanomol |
| Primaquine | PRIM, PQ | nanomol |
| Pyrimethamine | PYRIM | nanomol |
| Pyronaridine | PYRON | nanomol |
| Quinine | QIN, QN | nanomol |
| Quinazoline | QuiNazol | ng/ml |
| Trimethoprim | TRIMETHO | nanomol |
| Trimethotrexate | TRIMTX | nanomol |
| WR99210 | WR99210 | nanomol |
