# Supplementary material for: A Genome Wide Association Study of Plasmodium falciparum Susceptibility to 22 Antimalarial Drugs in Kenya
Source: PLoS One. 2014 May 8;9(5):e96486. doi: 10.1371/journal.pone.0096486 (PMC4014544; doi:10.1371/journal.pone.0096486)
Supplement: Table S2 — Amino acid haplotypes of hallmark variants in pfdhps and pfdhfr. Column ‘N’ is the number of samples in this study represented by that haplotype. (DOCX) [file pone.0096486.s012.docx]

| **DHPS** | | | **DHFR** | | | |  |
| --- | --- | --- | --- | --- | --- | --- | --- |
| **436** | **437** | **540** | **51** | **59** | **108** | **164** | **N** |
| A | A | K | I | R | N | I | 1 |
| S | A | K | I | C | N | I | 2 |
| S | A | K | I | R | N | I | 6 |
| S | A | K | I | R | N | L | 1 |
| S | G | E | I | C | N | I | 5 |
| S | G | E | I | R | N | I | 18 |
| S | G | E | N | R | N | I | 2 |
