# Supplementary material for: A Genome Wide Association Study of Plasmodium falciparum Susceptibility to 22 Antimalarial Drugs in Kenya
Source: PLoS One. 2014 May 8;9(5):e96486. doi: 10.1371/journal.pone.0096486 (PMC4014544; doi:10.1371/journal.pone.0096486)
Supplement: Table S3 — Variants detected in pfnhe. Column ‘N’ is the number of samples in this study carrying that allele. (DOCX) [file pone.0096486.s013.docx]

| **CHROM** | **POS** | **REF** | **ALT** | **AAC** | **N** |
| --- | --- | --- | --- | --- | --- |
| MAL13 | 175711 | T | C | K94R | 1 |
| MAL13 | 175367 | C | A | D209Y | 6 |
| MAL13 | 175360 | A | G | F211S | 1 |
| MAL13 | 175358 | A | C | Y212D | 1 |
| MAL13 | 175357 | T | G | Y212S | 2 |
| MAL13 | 175349 | C | T | D215N | 4 |
| MAL13 | 175346 | C | A | G216C | 4 |
| MAL13 | 173387 | G | A | H869Y | 2 |
| MAL13 | 173347 | T | C | K882R | 1 |
| MAL13 | 173310 | A | T | N894K | 4 |
| MAL13 | 172529 | C | T | D1155N | 1 |
| MAL13 | 171974 | C | A | A1340S | 1 |
| MAL13 | 171818 | T | C | N1392D | 1 |
| MAL13 | 171580 | T | C | K1471R | 2 |
| MAL13 | 171448 | C | A | R1515I | 1 |
