# Supplementary material for: A Genome Wide Association Study of Plasmodium falciparum Susceptibility to 22 Antimalarial Drugs in Kenya
Source: PLoS One. 2014 May 8;9(5):e96486. doi: 10.1371/journal.pone.0096486 (PMC4014544; doi:10.1371/journal.pone.0096486)
Supplement: Table S4 — Amino acid haplotypes of variants in pfcrt. Column ‘N’ is the number of samples in this study represented by that haplotype. (DOCX) [file pone.0096486.s014.docx]

| **Amino acid position** | | | | | | |  |
| --- | --- | --- | --- | --- | --- | --- | --- |
| **24** | **72** | **74** | **75** | **76** | **124** | **271** | **N** |
| D | C | I | E | T | R | E | 8 |
| D | C | M | N | K | Q | Q | 1 |
| D | C | M | N | K | R | Q | 17 |
| Y | C | M | N | K | R | Q | 2 |
