# Supplementary material for: A Genome Wide Association Study of Plasmodium falciparum Susceptibility to 22 Antimalarial Drugs in Kenya
Source: PLoS One. 2014 May 8;9(5):e96486. doi: 10.1371/journal.pone.0096486 (PMC4014544; doi:10.1371/journal.pone.0096486)
Supplement: Table S5 — Pairwise drug correlations. (DOCX) [file pone.0096486.s015.docx]

|  | WR99210 | LUM | ISOQIN | AMOD | DEAQ | PRIM | QIN | DHA | METHYLBL | MFL | PYRON | HLF | CQ | TRIMETHO | QuiNazol | PYRIM | PIQ | ATV | CHLOPROG | METHOT | CYCLOPG | TRIMTX |
| --- | --- | --- | --- | --- | --- | --- | --- | --- | --- | --- | --- | --- | --- | --- | --- | --- | --- | --- | --- | --- | --- | --- |
| WR99210 | 1 | 0.41 | 0.32 | 0.33 | 0.2 | -0.08 | -0.29 | 0.41 | -0.17 | 0.21 | 0.1 | 0.4 | 0.03 | 0.28 | 0.34 | 0.14 | -0.6 | -0.32 | -0.07 | -0.28 | -0.41 | -0.27 |
| LUM | 0.41 | 1 | 0.19 | -0.23 | -0.37 | 0.18 | 0.1 | 0.26 | 0.34 | 0.67 | -0.18 | 0.28 | -0.23 | 0.04 | 0.17 | 0.08 | -0.43 | -0.13 | 0.14 | -0.2 | 0.05 | -0.07 |
| ISOQIN | 0.32 | 0.19 | 1 | 0.42 | 0.55 | 0.07 | 0.36 | 0.46 | 0.32 | 0.46 | -0.13 | 0.25 | 0.28 | -0.23 | 0.14 | 0.01 | -0.34 | -0.13 | 0.15 | -0.08 | 0.03 | 0.06 |
| AMOD | 0.33 | -0.23 | 0.42 | 1 | 0.53 | -0.08 | -0.02 | 0.02 | -0.2 | -0.18 | 0.33 | 0.27 | 0.03 | 0.01 | 0.21 | 0.13 | -0.17 | 0.05 | -0.13 | 0.05 | 0.02 | 0.08 |
| DEAQ | 0.2 | -0.37 | 0.55 | 0.53 | 1 | -0.19 | -0.1 | 0.07 | -0.1 | -0.14 | 0.24 | 0.24 | 0.49 | 0.2 | 0.23 | 0.25 | -0.1 | 0.13 | 0.16 | 0.08 | 0.04 | 0.14 |
| PRIM | -0.08 | 0.18 | 0.07 | -0.08 | -0.19 | 1 | 0.21 | 0.15 | 0.32 | 0.23 | -0.09 | -0.11 | -0.48 | -0.26 | -0.02 | -0.11 | 0.25 | -0.01 | 0.43 | 0.17 | 0.22 | 0.05 |
| QIN | -0.29 | 0.1 | 0.36 | -0.02 | -0.1 | 0.21 | 1 | 0.18 | 0.51 | 0.46 | 0.06 | -0.06 | 0.12 | -0.32 | -0.1 | -0.05 | 0.04 | -0.02 | 0.18 | 0 | 0.35 | 0.52 |
| DHA | 0.41 | 0.26 | 0.46 | 0.02 | 0.07 | 0.15 | 0.18 | 1 | 0.59 | 0.62 | 0.02 | 0.3 | 0.06 | -0.08 | -0.06 | -0.11 | -0.02 | 0.13 | 0.4 | -0.04 | 0.11 | 0.13 |
| METHYLBL | -0.17 | 0.34 | 0.32 | -0.2 | -0.1 | 0.32 | 0.51 | 0.59 | 1 | 0.7 | 0.18 | 0.28 | 0.1 | -0.17 | -0.23 | -0.02 | 0.29 | 0.08 | 0.41 | 0.15 | 0.47 | 0.4 |
| MFL | 0.21 | 0.67 | 0.46 | -0.18 | -0.14 | 0.23 | 0.46 | 0.62 | 0.7 | 1 | 0.01 | 0.43 | 0.15 | -0.12 | -0.04 | 0.04 | -0.03 | 0.01 | 0.31 | -0.01 | 0.31 | 0.33 |
| PYRON | 0.1 | -0.18 | -0.13 | 0.33 | 0.24 | -0.09 | 0.06 | 0.02 | 0.18 | 0.01 | 1 | 0.67 | 0.06 | 0.33 | 0.1 | 0.33 | 0.47 | 0.34 | 0.13 | 0.28 | 0.4 | 0.65 |
| HLF | 0.4 | 0.28 | 0.25 | 0.27 | 0.24 | -0.11 | -0.06 | 0.3 | 0.28 | 0.43 | 0.67 | 1 | 0.12 | 0.32 | 0.31 | 0.42 | 0.21 | 0.14 | 0.18 | 0.26 | 0.33 | 0.43 |
| CQ | 0.03 | -0.23 | 0.28 | 0.03 | 0.49 | -0.48 | 0.12 | 0.06 | 0.1 | 0.15 | 0.06 | 0.12 | 1 | 0.3 | 0.18 | 0.29 | 0.13 | 0.18 | 0.09 | 0.06 | 0.12 | 0.18 |
| TRIMETHO | 0.28 | 0.04 | -0.23 | 0.01 | 0.2 | -0.26 | -0.32 | -0.08 | -0.17 | -0.12 | 0.33 | 0.32 | 0.3 | 1 | 0.67 | 0.81 | 0.15 | 0.13 | 0.3 | 0.38 | 0.14 | 0.16 |
| QuiNazol | 0.34 | 0.17 | 0.14 | 0.21 | 0.23 | -0.02 | -0.1 | -0.06 | -0.23 | -0.04 | 0.1 | 0.31 | 0.18 | 0.67 | 1 | 0.81 | 0.02 | 0.27 | 0.46 | 0.42 | 0.3 | 0.18 |
| PYRIM | 0.14 | 0.08 | 0.01 | 0.13 | 0.25 | -0.11 | -0.05 | -0.11 | -0.02 | 0.04 | 0.33 | 0.42 | 0.29 | 0.81 | 0.81 | 1 | 0.16 | 0.17 | 0.44 | 0.57 | 0.44 | 0.4 |
| PIQ | -0.6 | -0.43 | -0.34 | -0.17 | -0.1 | 0.25 | 0.04 | -0.02 | 0.29 | -0.03 | 0.47 | 0.21 | 0.13 | 0.15 | 0.02 | 0.16 | 1 | 0.49 | 0.44 | 0.43 | 0.5 | 0.45 |
| ATV | -0.32 | -0.13 | -0.13 | 0.05 | 0.13 | -0.01 | -0.02 | 0.13 | 0.08 | 0.01 | 0.34 | 0.14 | 0.18 | 0.13 | 0.27 | 0.17 | 0.49 | 1 | 0.46 | 0.39 | 0.58 | 0.45 |
| CHLOPROG | -0.07 | 0.14 | 0.15 | -0.13 | 0.16 | 0.43 | 0.18 | 0.4 | 0.41 | 0.31 | 0.13 | 0.18 | 0.09 | 0.3 | 0.46 | 0.44 | 0.44 | 0.46 | 1 | 0.3 | 0.53 | 0.44 |
| METHOT | -0.28 | -0.2 | -0.08 | 0.05 | 0.08 | 0.17 | 0 | -0.04 | 0.15 | -0.01 | 0.28 | 0.26 | 0.06 | 0.38 | 0.42 | 0.57 | 0.43 | 0.39 | 0.3 | 1 | 0.66 | 0.51 |
| CYCLOPG | -0.41 | 0.05 | 0.03 | 0.02 | 0.04 | 0.22 | 0.35 | 0.11 | 0.47 | 0.31 | 0.4 | 0.33 | 0.12 | 0.14 | 0.3 | 0.44 | 0.5 | 0.58 | 0.53 | 0.66 | 1 | 0.72 |
| TRIMTX | -0.27 | -0.07 | 0.06 | 0.08 | 0.14 | 0.05 | 0.52 | 0.13 | 0.4 | 0.33 | 0.65 | 0.43 | 0.18 | 0.16 | 0.18 | 0.4 | 0.45 | 0.45 | 0.44 | 0.51 | 0.72 | 1 |
