# Supplementary material for: A Genome Wide Association Study of Plasmodium falciparum Susceptibility to 22 Antimalarial Drugs in Kenya
Source: PLoS One. 2014 May 8;9(5):e96486. doi: 10.1371/journal.pone.0096486 (PMC4014544; doi:10.1371/journal.pone.0096486)
Supplement: Table S6 — European Nucleotide Archive accession numbers and corresponding phenotypic data for the 35 samples used in this study. Drug abbreviations and concentration units are described in table S1. (DOCX) [file pone.0096486.s016.docx]

| **Accession** | **CQ** | **MFL** | **HLF** | **LUM** | **PIQ** | **QIN** | **PRIM** | **QuiNazol** | **AMOD** | **DEAQ** | **ISOQIN** | **PYRON** | **METHYLBL** | **DHA** | **METHOT** | **TRIMTX** | **PYRIM** | **TRIMETHO** | **CHLOPROG** | **CYCLOPG** | **ATV** | **WR99210** |
| --- | --- | --- | --- | --- | --- | --- | --- | --- | --- | --- | --- | --- | --- | --- | --- | --- | --- | --- | --- | --- | --- | --- |
| ERS016375 | 30.725 | 40.27175 | 92.505 | 169.28 | 36.28 | 122.345 | 1903.525 | 10.5665 | 7.79 | 17.43 | 3.7 | 34.85 | 7.8998 | 1.855 | 113.275 | 17.1174 | 2175.92 | 17394.313 | 4667.6 | 23 | 0.7 | 1.5329 |
| ERS010455 | 90.705 | 42.05585 | 11.275 | 104.155 | 30.645 | 78.155 | 394.87 | 18.6265 | 5.475 | 8.515 | 2.18 | 4.325 | 5.7025 | 0.855 | 128.75 | 15.69085 | 2765.46 | 19404.3195 | 4602.09 | 38.035 | 2.24 | 0.28905 |
| ERS010435 | 11.04 | 15.79805 | 10.6 | 93.375 | 30.985 | 33.73 | 458.2 | 6.3761 | 3.815 | 5.74 | 2.11 | 4.675 | 2.01295 | 1.04 | 147.355 | 5.71765 | 917.435 | 11735.093 | 830.595 | 10.995 | 1.31 | 0.31265 |
| ERS010454 | 49.015 | 21.75495 | 7.965 | 123.575 | 26.92 | 27.825 | 1755.445 | 4.4796 | 2.615 | 3.915 | 1.43 | 3.085 | 3.01325 | 1.43 | 127.975 | 4.76005 | 1041.71 | 10088.879 | 4631.925 | 17.44 | 2.36 | 0.23785 |
| ERS010464 | 83.05 | 3.22695 | 0.675 | 22.945 | 32.65 | 55.815 | 443.725 | 3.44875 | 6.23 | 9.385 | 2.175 | 1.51 | 0.9996 | 0.59 | 108.59 | 5.5181 | 479.82 | 4086.035 | 2308.06 | 7.08 | 1.7 | 0.128 |
| ERS017458 | 41.575 | 40.19575 | 34.53 | 238.83 | 42.815 | 268.27 | 606.56 | 29.3145 | 7 | 7.245 | 3.75 | 5.81 | 9.26965 | 0.675 | 160.405 | 30.9052 | 2511.43 | 6268.2795 | 4067.38 | 57.545 | 0.775 | 0.0076 |
| ERS016368 | 80.14 | 40.8371 | 71.9 | 97.635 | 24.795 | 198.595 | 623.14 | 23.2465 | 9.75 | 20.5 | 8.025 | 33.865 | 9.5151 | 1.93 | 88.32 | 22.7099 | 1562.23 | 10445.1625 | 7803.55 | 30.435 | 1.67 | 0.92305 |
| ERS010641 | 73.015 | 9.01225 | 18.505 | 39.365 | 24.2 | 50.87 | 207.835 | 2.1535 | 9.675 | 16.675 | 3.495 | 10.845 | 1.5914 | 1.36 | 122.225 | 9.60425 | 1082.535 | 9515.328 | 973.545 | 11.895 | 0.29 | 0.40445 |
| ERS016369 | 30.18 | 33.34135 | 27.305 | 69.345 | 20.925 | 202.775 | 484.19 | 4.363 | 7.665 | 7.425 | 2.885 | 11.585 | 2.327 | 1.425 | 101.455 | 11.27575 | 584.865 | 2963.924 | 2096.55 | 8.23 | 0.27 | 0.95345 |
| ERS017460 | 31.61 | 36.7191 | 27.47 | 59.53 | 23.48 | 192 | 850.9 | 2.92 | 10.04 | 10.24 | 5.06 | 5.11 | 3.5172 | 0.98 | 171.98 | 13.4102 | 652.12 | 863.54 | 586.77 | 41.9 | 1.62 | 0.0025 |
| ERS017459 | 83.37 | 33.914 | 16.86 | 123.165 | 17.475 | 207.235 | 326.705 | 3.6375 | 6.615 | 5.745 | 2.105 | 5.315 | 0.95855 | 0.47 | 74.035 | 7.15445 | 938.695 | 9971.096 | 544.465 | 14.03 | 0.435 | 0.66565 |
| ERS017455 | 32.595 | 35.6631 | 10.52 | 60.26 | 18.21 | 262.77 | 1605.61 | 0.4965 | 8.49 | 8.46 | 3.465 | 18.98 | 4.5911 | 1.1 | 110.91 | 24.64135 | 446.02 | 1725.2375 | 1600.905 | 11.025 | 1.675 | 0.79115 |
| ERS016376 | 109.72 | 53.5502 | 89.08 | 174.4 | 63.64 | 154.28 | 2154.58 | 20.534 | 9.31 | 13.43 | 6.22 | 23.35 | 15.3442 | 2.87 | 178.82 | 18.6319 | 1887.36 | 9864.529 | 4808.97 | 56.86 | 5.46 | 0.9129 |
| ERS010401  ERS010402 | 50.125 | 17.11195 | 18.6 | 46.22 | 38.63 | 129.98 | 311.355 | 0.0142 | 9.99 | 8.59 | 2.25 | 29.98 | 8.66955 | 1.42 | 95.115 | 12.0291 | 550.895 | 2453.7405 | 1715.16 | 26.64 | 2.485 | 0.0213 |
| ERS010403 | 12.99 | 5.3674 | 40.035 | 49.895 | 89.365 | 42.375 | 591.025 | 58.04855 | 8.405 | 8.64 | 1.295 | 45.7 | 0.1033 | 0.425 | 204.425 | 38.26965 | 4842.31 | 40546.7055 | 4568.83 | 93.04 | 19.825 | 0.01115 |
| ERS010407  ERS010408 | 56.405 | 17.39265 | 89.455 | 90.81 | 56.885 | 32.795 | 240.125 | 3.78815 | 7.88 | 8.53 | 2.185 | 42.06 | 1.2626 | 0.2 | 78.51 | 12.90195 | 873.245 | 9687.469 | 1012.76 | 10.65 | 2.8 | NA |
| ERS010409  ERS010410 | 76.03 | 7.8687 | 40.745 | 30.875 | 68.045 | 54.07 | 418.31 | 33.88525 | 8.555 | 9.785 | 1.37 | 40.18 | 0.14485 | 0.125 | 156.695 | 20.78705 | 2534.25 | 19865.7175 | 3278.8 | 38.805 | 8.345 | NA |
| ERS010451 | 110.31 | 30.9385 | 36.195 | 50.185 | 19.8 | 181.54 | 242.125 | 27.01775 | 8.975 | 18.435 | 3.465 | 36.315 | 3.30645 | 1.07 | 165.715 | 49.75945 | 3796.325 | 18756.329 | 3218.945 | 107.84 | 4.69 | 0.43135 |
| ERS010638 | 95.93 | 40.5743 | 41.47 | 115.96 | 17.13 | 67.68 | 282.595 | 3.2355 | 7.85 | 22.59 | 6.635 | 13.57 | 1.9529 | 1.2 | 58.37 | 9.6773 | 615.61 | 5825.9985 | 509.905 | 8.53 | 1.095 | 1.19815 |
| ERS016370 | 78.965 | 63.86915 | 19.74 | 274.505 | 23.07 | 286.945 | 385.51 | 29.9905 | 6.505 | 6.53 | 4.18 | 4.24 | 3.3655 | 1.495 | 104.53 | 20.68515 | 1319.21 | 5734.073 | 3979.365 | 37.825 | 7.525 | 0.26375 |
| ERS010411  ERS010412 | 92.955 | 52.9172 | 24.21 | 97.84 | 82.565 | 270.19 | 328.59 | 2.07865 | 6.375 | 7.91 | 2.5 | 26.005 | 25.80965 | 1.72 | 123.34 | 30.9761 | 777.255 | 16545.348 | 2389.96 | 37.8 | 1.99 | 0.00215 |
| ERS010457 | 86.16 | 17.5761 | 14.91 | 36.76 | 34.91 | 145.77 | 414.345 | 3.94205 | 8.135 | 16.99 | 3.405 | 23.975 | 2.7722 | 0.84 | 139.385 | 11.49305 | 1314.605 | 9796.1825 | 922.155 | 13.57 | 2.595 | 0.06125 |
| ERS010413  ERS010414 | 114.67 | 3.5479 | 5.33 | 33.43 | 56.44 | 238.76 | 542.54 | 7.088 | 7.86 | 21.67 | 3.4 | 16.49 | 1.3984 | 0.33 | 130.18 | 49.9524 | 932.9 | 7645.151 | 4301.14 | 52.7 | 3.9 | 0.0021 |
| ERS010461 | 54.935 | 20.34035 | 5.45 | 75.09 | 28.87 | 91.615 | 576.885 | 0.00715 | 3.41 | 8.63 | 2.275 | 4.215 | 5.34225 | 0.475 | 63.535 | 4.0462 | 245.945 | 1440.326 | 1659.36 | 20.715 | 1.355 | 5e-05 |
| ERS010452 | 14.98 | 31.4788 | 9.455 | 84.595 | 56.195 | 207.675 | 4305.555 | 0 | 7.905 | 8.51 | 3.485 | 9.17 | 6.578 | 1.2 | 83.595 | 6.9019 | 128.6 | 897.3005 | 4147.21 | 41.145 | 3.095 | 0 |
| ERS016371 | 14.02 | 16.0402 | 10.37 | 284.74 | 20.92 | 81.96 | 467.18 | 5.126 | 11.52 | 8.1 | 2.11 | 12.8 | 0.5971 | 0.93 | 66.86 | 7.6363 | 840.1 | 9230.67 | 297.68 | 10.59 | 1.17 | 0.9154 |
| ERS010462 | 87.195 | 36.1515 | 45.1 | 103.515 | 41.96 | 233.955 | 277.06 | 5.3539 | 6.425 | 11.145 | 2.885 | 45.255 | 26.1194 | 3.225 | 140.575 | 59.1883 | 1458.395 | 9990.156 | 4418.98 | 95.89 | 12.445 | 0.23345 |
| ERS016372 | 16.765 | NA | NA | 346.385 | 12.145 | NA | NA | NA | NA | NA | NA | NA | NA | 1.59 | NA | NA | NA | NA | NA | NA | NA | NA |
| ERS010644 | 10.11 | NA | NA | 332.435 | 16.15 | NA | NA | NA | NA | NA | NA | NA | NA | 0.45 | NA | NA | NA | NA | NA | NA | NA | NA |
| ERS010416  ERS010415 | 35.33 | 34.9506 | 21.2 | 59.25 | 82.29 | 381.8 | 1748.64 | 0.0383 | 4.27 | 3.21 | 1.16 | 49.7 | 12.537 | 0.07 | 138.98 | 50.5258 | 1391.69 | 7271.692 | 1914.76 | 52.83 | 1.02 | 6e-04 |
| ERS010453 | 16.84 | 71.5203 | 41.82 | 130.02 | 70.5 | 81.18 | 730.45 | 0.0036 | 4.94 | 3.05 | 1.74 | 35.09 | 9.7399 | 2.42 | 130.11 | 48.0824 | 99.85 | 215.963 | 2741.8 | 101.37 | 10.03 | 0 |
| ERS016373 | 2.345 | NA | NA | 225.13 | 15.075 | NA | NA | NA | NA | NA | NA | NA | NA | 0.995 | NA | NA | NA | NA | NA | NA | NA | NA |
| ERS010640 | 112.98 | NA | NA | 410.535 | 19.675 | NA | NA | NA | NA | NA | NA | NA | NA | 0.76 | NA | NA | NA | NA | NA | NA | NA | NA |
| ERS016374 | 2.27 | NA | NA | 371.765 | 18.675 | NA | NA | NA | NA | NA | NA | NA | NA | 0.49 | NA | NA | NA | NA | NA | NA | NA | NA |
| ERS010438 | 15.705 | 39.35435 | 9.535 | 67.115 | 56.685 | 270.42 | 1516.52 | 0.1584 | 6.805 | 8.8 | 3.46 | 8.095 | 4.5459 | 1.58 | 110.055 | 43.2382 | 687.22 | 2263.0595 | 5149.99 | 30.6 | 2.96 | 1e-04 |
